# History of antineutrophil cytoplasmic autoantibodies: Milestones in rheumatology

**DOI:** 10.1007/s00393-024-01599-4

**Published:** 2024-12-10

**Authors:** Kirsten de Groot, Elena Csernok, Diane van der Woude

**Affiliations:** 1 3rd Medical Department of Nephrology, Internal Medicine, Nephrology, Rheumatology, Sana Klinikum Offenbach, KfH Nierenzentrum Offenbach, Starkenburgring 66, 63069 Offenbach, Germany; 2 Department Internal Medicine, Nephrology, Rheumatology. Medius Kliniken, Kirchheim-Teck, Germany; 3 Department of Rheumatology, Leiden University Medical Center, Leiden, The Netherlands

**Keywords:** PR3-ANCA, MPO-ANCA, Antigen specific immunoassay, Necrotizing small-vessel vasculitides, History, PR3-ANCA, MPO-ANCA, Antigen-spezifischer Immunoassay, Nekrotisierende Kleingefäßvaskulitiden, Geschichte

## Abstract

Antineutrophil cytoplasmic antibody (ANCA)-associated vasculitides (AAV) are autoimmune inflammatory small-vessel disorders with potentially life-threatening organ manifestations. Recent disease definitions and classification criteria allow distinction between granulomatosis with polyangiitis (GPA), eosinophilic granulomatosis with polyangiitis (EGPA), and non-granulomatous microscopic polyangiitis (MPA). The discovery of ANCA—autoantibodies directed against proteolytic enzymes of neutrophil granules—has enabled earlier diagnosis of AAV and paved the way to stage-adapted treatments. ANCA testing initially relied on different immunofluorescence patterns, i.e., cytoplasmic ANCA (C-ANCA) vs. perinuclear ANCA (P-ANCA), in ethanol-fixed neutrophils. This is nowadays outperformed by well-standardized immunoassays against the ANCA target antigens proteinase 3 (PR3) and myeloperoxidase (MPO) for the diagnosis of small-vessel vasculitides. The discovery of ANCA has contributed substantially to unravelling the pathogenesis of AAV, which comprises neutrophil degranulation, NETosis with concurrently amplified ANCA antigen presentation, and intra- and transmural vascular inflammation involving the alternative complement system in susceptible individuals. There is a genetic disposition concerning certain HLA alleles and polymorphisms of the proteinase 3 gene. Furthermore, epigenetic modifications impact on disease activity and relapse. During follow-up, the ANCA titer is not a reliable mirror of disease activity; however, PR3-ANCA positivity is associated with a greater likelihood of relapse and a better treatment response to rituximab as compared to cyclophosphamide/azathioprine. Within the past 60 years, the discovery of ANCA has revolutionized the ability to diagnose, understand, classify, and treat AAV in a targeted manner.

## Abstract (de)

Mit antineutrophilen zytoplasmatischen Antikörpern (ANCA) assoziierte Vaskulitiden (AAV) sind autoimmun bedingte, entzündliche Erkrankungen kleiner Gefäße mit potenziell lebensbedrohlichen Organmanifestationen. Neue Krankheitsdefinitionen und Klassifikationskriterien erlauben die Abgrenzung der Granulomatose mit Polyangiitis (GPA) von der eosinophilen Granulomatose mit Polyangiitis sowie der mikroskopischen Polyangiitis (MPA). Die Entdeckung der ANCA, gegen Enzyme aus neutrophilen Granula gerichtete Autoantikörper, hat die frühzeitige Diagnose der AAV ermöglicht und den Weg zur stadienadaptierten Therapie geebnet. Die initiale ANCA-Testung beruhte auf unterschiedlichen Immunfluoreszenzmustern – C‑ (zytoplasmatische) ANCA vs. P‑ (perinukleäre) ANCA – auf ethanolfixierten neutrophilen Granulozyten. Diese Technik wird heute durch gut standardisierte Immunoassays gegen die ANCA-Target-Antigene Proteinase 3 (PR3) und Myeloperoxidase (MPO) im Kontext der Diagnostik von Kleingefäßvaskulitiden übertroffen. Die Entdeckung der ANCA hat maßgeblich zur Offenlegung der Pathogenese der AAV beigetragen, die die Degranulation aktivierter neutrophiler Granulozyten, NETose mit konsekutiver verstärkter Antigenpräsentation, intra- und transmuraler vaskulärer Entzündung unter Einbeziehung des alternativen Komplementwegs bei hierfür empfänglichen Personen umfasst. Es besteht eine genetische Prädisposition bezüglich bestimmter HLA-Allele sowie Polymorphismen im Proteinase-3(PR3)-Gen. Darüber hinaus beeinflussen epigenetische Modifikationen Krankheitsaktivität und Rezidivhäufigkeit. Im longitudinalen Krankheitsverlauf spiegelt der ANCA-Titer nicht zuverlässig die Krankheitsaktivität wider, jedoch ist eine persistierende PR3-ANCA-Positivität mit einer größeren Wahrscheinlichkeit eines Rezidivs und einem besseren Ansprechen auf eine Therapie mit Rituximab im Vergleich zu Cyclophosphamid/Azathioprin verbunden. Die Entdeckung der ANCA hat in den letzten 60 Jahren die Möglichkeit revolutioniert, AAV zu diagnostizieren, zu klassifizieren, zu verstehen und gezielt zu behandeln.

## Introduction

The detection and characterization of antineutrophil cytoplasmic autoantibodies (ANCA) have revolutionized the diagnostic accuracy of potentially life-threatening necrotizing small-vessel vasculitides, termed ANCA-associated vasculitides (AAV). ANCA are directed against intracellular constituents of neutrophil granulocytes and monocytes and induce characteristic fluorescence patterns in indirect immunofluorescence (IIF) in ethanol-fixed neutrophils. There are two main fluorescence patterns on the neutrophil substrate: the cytoplasmic pattern (C-ANCA) or perinuclear staining (P-ANCA). The relevant autoantigens for C‑ and P‑ANCA were identified as proteinase 3 and myeloperoxidase, respectively (PR3- and MPO-ANCA).

AAV comprise granulomatosis with polyangiitis (GPA), microscopic polyangiitis (MPA), and eosinophil granulomatosis with polyangiitis (EGPA). Undiagnosed and untreated, most courses of GPA and MPA, the most frequent AAV, result in ultimately fatal pulmonary-renal syndrome. The discovery of ANCA as a diagnostic tool has allowed the accurate distinction of ANCA-associated vasculitis from other diseases and thus enabled a proper estimation of the true incidence of the diseases as well as recognition of earlier disease stages. This paved the way for stage- and disease-extent-adapted therapies.

This paper aims at reviewing the history of detection of ANCA and the development of standardized diagnostic tools as well as discussing the predictive value of these markers in the disease course and classification of AAV.

## How ANCA started

### ANCA-associated vasculitis—from names to acronyms

At the time when ANCA were first described, the nomenclature of the diseases was different from what it is today. Wegener’s granulomatosis (after Friedrich Wegener) is now called granulomatosis with polyangiitis (GPA). Churg–Strauss syndrome (after Jacob Churg and Lotte Strauss—one of the few diseases to be named after a woman) is now known as eosinophilic granulomatosis with polyangiitis (EGPA). Polyarteriitis nodosa was used to denote both the medium-sized vessel vasculitis now designated as PAN (panarteriitis nodosa) and the small-vessel vasculitis, currently called MPA. For the sake of clarity, we will refer to the diseases by their current acronyms in this manuscript.

### Intriguing patterns in immunofluorescence

The history of the discovery of ANCA, especially the development of the detection methods, encompassed several decades. Autoantibodies against polymorphonuclear leukocytes were discovered in 1959 in several chronic inflammatory disorders [1] and the association between vasculitis, in particular glomerulonephritis, and these autoantibodies reacting to cytoplasmic components of neutrophils, was described in [2]. In 1985 similar autoantibodies termed anti-cytoplasmic antibodies (ACPA) were detected by indirect immunofluorescence (IIF) in ethanol-fixed neutrophil granulocytes in a mixed Dutch/Danish cohort of GPA patients [3]. Using a large cohort of disease controls, the specificity of ACPA for GPA was established, as well as the absence of this autoantibody in early stages with limited disease manifestations and in disease remission [4].

### From patterns to enzymes

**Fig. 1:**
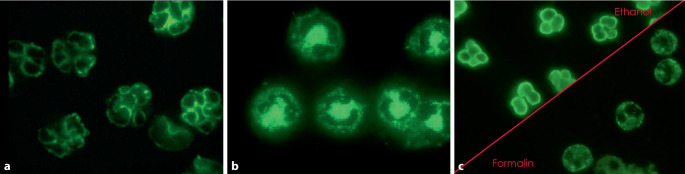
**a**, **b** Main patterns of antineutrophil cytoplasmic autoantibody (ANCA) staining. **a** Typical P‑ANCA staining pattern of ethanol-fixed neutrophils with P‑/MPO-ANCA-positive serum; **b** C-ANCA staining pattern of ethanol-fixed neutrophils with C‑/PR3-ANCA-positive serum, **c** Formalin fixation enables C‑ and P‑ANCA to be distinguished. Formalin fixation prevents translocation of basic (positively charged) proteins, such as MPO, into the negatively charged nucleus (image by AESCU-DIAGNOSTIC)

In 1989, American renal scientists described two different IIF patterns in ethanol-fixed neutrophils in a cohort of patients with crescentic and/or necrotizing glomerulonephritis with (GPA, MPA) and without extrarenal symptoms: a *cytoplasmic* and a *perinuclear *staining pattern. The perinuclear/nuclear staining pattern (later P‑ANCA; Fig. 1a) was shown to correlate with reactivity to the enzyme myeloperoxidase (MPO) from neutrophil alpha granules in ELISA, as established by subcellular neutrophil fragmentation experiments [5]. In contrast, most of the ACPA-positive sera from patients with necrotizing crescentic glomerulonephritis (NCGN), which did not react with MPO, displayed a cytoplasmic fluorescence pattern on IIF (later C‑ANCA; Fig. 1b). Fixation of cells by ethanol allows discrimination between the difference fluorescence pattern (C- or P‑ANCA). Interestingly, it was demonstrated that the P‑ANCA pattern is an artefact of ethanol fixation (Fig. 1c). The relevant target antigen of ACPA in patients with GPA was identified as a 29 KD lysosomal serine protease in myeloid cells by several groups [6–8]. It was sequenced and proven to be identical to proteinase 3 [9]. The specificity of ACPA for GPA in IIF and ELISA was found to be 97 to 99%, respectively; sensitivity depended on disease stage and was 95% in generalized active disease in a large cohort of GPA patients as compared to controls [10].

## Standardization of ANCA testing

At the second international ANCA workshop 1989 in the Netherlands, ACPA was renamed as ANCA. ANCA detection was determined as a two-step process with initial IIF assay in ethanol-fixed neutrophils and confirmation of positive samples by antigen-specific immunoassays (PR3- and/or MPO-ANCA). The multicenter studies of Hagen et al. paved the way for an international consensus on the appropriate detection of ANCA in patients suspected of AAV [11–13]. Those international consensus guidelines dictated that screening for ANCA should be performed by IIF in ethanol-fixed neutrophils, and positive results were to be confirmed by specific immunoassays for PR3- and/or MPO-ANCA [14].

Since publication of the international consensus on ANCA detection, many new developments (computer-based image analysis of immunofluorescence patterns, novel antigen specific assays) have emerged. Next to the original PR3-/MPO-ANCA ELISA, novel solid-phase technologies, like addressable laser bead immunoassays (ALBIA), chemiluminescent immunoassays (CLIA), fluorescent-enzyme immunoassays (FEIA), line or dot immunoassays (LIA/DIA), and even IIF by image analysis, have become available. In addition, antigen binding to the solid phase has evolved from direct binding towards binding via a capturing monoclonal antibody or via a peptide linker, i.e., first-, second-, and third-generation ANCA assays, respectively [15].

**Fig. 2:**
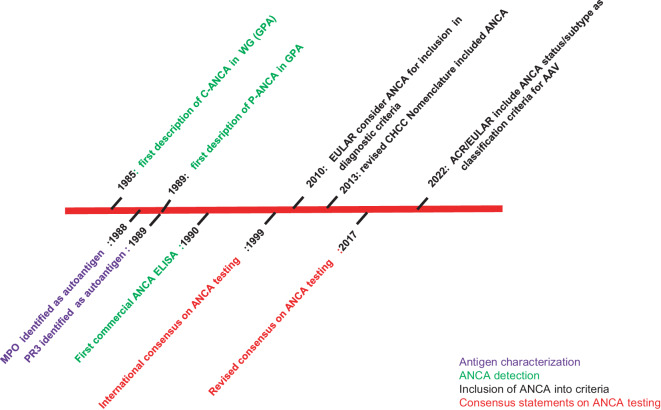
Chronological overview of the development of antineutrophil cytoplasmic autoantibodiy (ANCA) testing

Recently, based on a multicenter study by the European Vasculitis Study Group (EUVAS) in 2017, the diagnostic value of PR3- and MPO-ANCA immunoassays was found to be equal to or even exceed the diagnostic performance of IIF [16]. Consequently, these findings have precipitated in a revised consensus on ANCA testing for AAV ([17]; Fig. 2). This new consensus states that high-quality antigen-specific immunoassays should be used as the primary screening method in patients with suspected AAV, without the need for IIF. However, the current consensus recommendation applies to ANCA testing for the diagnosis of small-vessel vasculitis. Furthermore, infections should be ruled out (e.g., PR3-ANCA positivity in patients with infectious endocarditis), and a detailed history of medications and illicit drug should use be retrieved.

The performance of this consensus recommendation should be evaluated prospectively.

However, it is important to note that this recommendation does not apply to the setting of, e.g., IBD, autoimmune hepatitis, and drug- and cocaine-induced autoimmunity, in which so-called atypical ANCA exist which are positive in IIF but mostly bind to other antigens such as neutrophil elastase, bactericidal permeability-increasing protein, cathepsin G, or lactoferrin.

## The role of ANCA in disease classification: ANCA status or clinical phenotype

In parallel with the establishment of ANCA as a diagnostic tool, classification criteria for primary vasculitides [18] were established in order to enable the separation of GPA from other small-vessel vasculitides (EGPA and polyarteritis nodosa), at this stage relying on clinical symptoms and histology only. MPA was not recognized as a distinct entity at the time. This was followed by disease definitions for vasculitides, in which MPA was defined as an entity separate from GPA, EGPA, and polyarteritis nodosa [19].

Only recently, ACR/EULAR set up new weighted classification criteria for GPA, MPA, and EGPA that also include the ANCA status/subtype and imaging results [20–22]. The fact that the presence of PR3-ANCA in GPA and MPO-ANCA in MPA is endowed with the highest number of points (5 and 6, respectively), whereas positive MPO-ANCA in GPA and positive PR3-ANCA in MPA each result in one negative point attests to the crucial role of these antibodies in diagnosing GPA and MPA. For EGPA on the other hand, it is worthwhile noting that the presence of ANCA yields minus points, indicating that it is harder to fulfil the classification criteria when these antibodies are present. This is in line with the much lower prevalence of ANCA in EGPA (approximately 40%) as compared to GPA and MPA, and it is due to the fact that these criteria were specifically developed to distinguish these diseases from one another.

The key importance of ANCA as a marker distinguishing between different AAV entities is also poignantly illustrated by the findings of genetic association studies. The strongest associations of genetic factors were found with the antigenic specificity of ANCA (PR3 versus MPO) and not with the phenotypic clinical syndromes GPA or MPA [23]. This indicates that ANCA are not just surrogate markers useful for diagnostic purposes, but that they are most likely directly involved in the causal pathophysiological pathways underlying the disease. Another genetic observation supporting this is the association between anti-PR3-ANCA and genetic polymorphisms in the proteinase 3 gene, again suggesting that the autoimmune response against this antigen is a feature central to the pathogenic process.

## Role of ANCA in the pathogenesis of AAV

Decades of experimental research using both human material and several elegant rodent models have collectively led to the following current model of the role of ANCA in the immunopathogenesis of small-vessel vasculitis [24]:

In individuals predisposed to ANCA vasculitis due to genetic risk factors, such as certain HLA alleles or PR3 polymorphisms, tolerance to PR3 and MPO is broken by antigen-presenting cells presenting HLA-bound peptides derived from these molecules to T cells, which are thereby activated. These T cells provide help to PR3- or MPO-reactive B cells, leading to the emergence of anti-PR3 and anti-MPO antibodies: a phenomenon that by itself is not sufficient to lead to vasculitis. In normal circumstances, the PR3 and MPO molecules are located in the cytoplasm of neutrophils, and it is only upon activation (“priming”) of neutrophils by, e.g., inflammatory cytokines, that these antigens relocate to the neutrophil cell surface (PR3 does so via binding to CD177; MPO is expressed on the cell surface to a lesser extent). Circulating ANCA can then bind to their antigens with their F(ab)2 parts, while at the same time their Fc part can engage Fcγ receptors on the neutrophil cell surface, which leads to further triggering of these cells. Activated neutrophils then secrete various cytotoxic molecules, including reactive oxygen species, and neutrophil extracellular traps (i.e., undergo NETosis): processes that lead to damage of the endothelium. Concurrently, the alternative complement pathway is activated, leading to the recruitment of more neutrophils to the ongoing inflammation and resulting in a vicious cycle of inflammation, ultimately leading to tissue necrosis.

Furthermore, epigenetic modifications are associated with disease activity in AAV. Hypomethylation in certain regions of the *PR3* and *MPO* genes was found in active disease, whereas in remission, DNA methylation was increased, leading to a reduced expression of the respective autoantigen and a reduced likelihood of relapse in GPA [25]. In addition, aberrant histone modification profiles were found to be involved in GPA, MPA, and EGPA.

## ANCA as a biomarker in the management of AAV

In a recent meta-analysis [26], the solid-phase assays for PR3 and MPO displayed a reasonable sensitivity and a high specificity for the diagnosis of AAV. This means that a negative PR3- or MPO-ANCA test almost certainly excludes the disease, at least in a generalized stage. An important precondition of ordering an ANCA test is, however, to enhance the pretest probability by using a clinical gating strategy, namely only testing patients with a certain set of clinical symptoms and findings (see Table 1).

**Table 1:** Gating strategy for using solid-phase assays for Proteinase 3 (PR3) and myeloperoxidase (MPO) for the diagnosis of antineutrophil cytoplasmic antibody (ANCA)-associated vasculitides (AAV) (extended from Bossuyt Nature Rev Rheumatology [17])

Glomerulonephritis, especially rapidly progressive glomerulonephritis
Pulmonary hemorrhage, especially pulmonary-renal syndrome
Interstitial lung disease
Cutaneous vasculitis with systemic features
Multiple lung nodules
Chronic destructive disease of the upper airways
Long-standing sinusitis or otitis media
Subglottic tracheal stenosis
Mononeuritis multiplex or other peripheral neuropathy
Retro-orbital mass
Scleritis

Many cohort studies have proven that relapses are more frequently associated with PR3- than with MPO-ANCA [27, 28], irrespective of the treatment modality.

By contrast, the clinical utility of serial measurements of ANCA titers in the disease course for prediction of remission or relapse in individual patients remains elusive. Patients with ANCA conversion from seropositive to seronegative under cyclophosphamide and methotrexate induction therapy experienced fewer relapses than those without. However, in a meta-analysis of 15 studies, a rise or persistence of PR3- or MPO-ANCA had only a moderate predictive value for future relapse [29]. Furthermore, variation in ANCA titer (increase, decrease) was not associated with time to remission or relapse [30].

Trials using rituximab for induction and/or maintenance of remission [31, 32] achieved a lower relapse rate with rituximab than with conventional cytotoxic treatment (sequence of cyclophosphamide/azathioprine), especially in PR3-positive patients with first diagnosis or relapse of the disease. However, there was no difference in treatment response to rituximab between the clinical phenotypes of GPA versus MPA [33]. In addition, ANCA conversion occurred more often with rituximab in PR3-positive patients as compared to with cyclophosphamide, whereas there was no such difference in MPO-positive patients.

A rise in PR3-ANCA titer after achieving complete remission with rituximab was moderately associated with a future relapse, especially in patients with initial presentation of alveolar hemorrhage or renal involvement [34, 35]. There is no clinically meaningful association with a rise in MPO-ANCA, regardless of the treatment regimen, or of a rise in MPO- or PR3-ANCA with cyclophosphamide/azathioprine.

As the likelihood of achieving remission and not experiencing a relapse in PR3-ANCA-positive disease is higher with rituximab than with cyclophosphamide/azathioprine, the former is the preferred induction and maintenance regimen for these patients according to recent guidelines for the management of AAV [36, 37].

However, during the disease course, it is not advised to take treatment decisions based on the course of the PR3- or MPO-ANCA titer alone.

In conclusion, the discovery of ANCA has transformed the diagnosis, classification, pathophysiological insights, and treatment algorithms in small-vessel vasculitis. The history of the development of ANCA testing is a marvelous example of research from bench to bedside, having opened up a completely new field of research for an international scientific community. This would not have been possible without the groundbreaking work of Prof. Dr. Wolfgang Gross and Prof. Dr. Fokko van der Woude. We are indebted to their legacy and happy to be part of such a great community.

## Declarations

For this article no studies with human participants or animals were performed by any of the authors. All studies mentioned were in accordance with the ethical standards indicated in each case.
